# A prediction model for the impact of environmental and genetic factors on cardiovascular events: development in a salt substitutes population

**DOI:** 10.1186/s12967-023-03899-w

**Published:** 2023-01-30

**Authors:** Dan Zhao, Hao Sun, Huamin Li, Chaoxiu Li, Bo Zhou

**Affiliations:** 1 Department of Clinical Epidemiology and Evidence-Based Medicine, The First Hospital of China Medical University, No.155, Nanjing North Street, Heping District, Shenyang, Liaoning China; 2 School of Public Health, China Medical University, Shenyang, Liaoning China

**Keywords:** CV events, Copy number variations, Prediction model, Predicted probability, Low-sodium salt

## Abstract

**Background:**

Cardiovascular disease (CVD) has evolved into a serious public health issue that demands the use of suitable methods to estimate the risk of the disease. As a result, in a sample of individuals who completed a 3-year low-sodium salt or conventional salt intervention in a hypertensive environment, we constructed a 13-year cardiovascular (CV) event risk prediction model with a 10-year follow-up.

**Methods:**

A Cox proportional hazards model was used to build a prediction model based on data from 306 participants who matched the inclusion criteria. Both the discriminating power and the calibration of the prediction models were assessed. The discriminative power of the prediction model was measured using the area under the curve (AUC). Brier scores and calibration plots were used to assess the prediction model's calibration. The model was internally validated using the tenfold cross-validation method. The nomogram served as a tool for visualising the model.

**Results:**

Among the 306 total individuals, there were 100 cases and 206 control. In the model, there were six predictors including age, smoking, LDL-C (low-density lipoprotein cholesterol), baseline SBP (systolic blood pressure), CVD (cardiovascular history), and CNV (genomic copy number variation) nsv483076. The fitted model has an AUC of 0.788, showing strong model discrimination, and a Brier score of 0.166, indicating that it was well-calibrated. According to the results of internal validation, the prediction model utilised in this study had a good level of repeatability. According to the model integrating the interaction of CNVs and baseline blood pressure, the effect of baseline SBP on CV events may be greater when nsv483076 was normal double copies than when nsv483076 was copy number variation.

**Conclusions:**

The efficacy of risk prediction models for CV events that include environmental and genetic components is excellent, and they may be utilised as risk assessment tools for CV events in specific groups to offer a foundation for tailored intervention strategies.

**Supplementary Information:**

The online version contains supplementary material available at 10.1186/s12967-023-03899-w.

## Background

Globally, the number of fatalities due to cardiovascular disease (CVD) has grown by a quarter since 2000, reaching 17.9 million in 2019. CVD has become a major public health issue owing to its rising economic costs. With complex comorbidity, CVD takes a long time to develop. As a result, a proper strategy is necessary to forecast the development of cardiovascular (CV) events and give effective intervention to avert them. Researchers have built various CVD prediction models over the last few decades, and they've employed statistical approaches to incorporate multiple factors to estimate CVD risk. Framingham [1–3], SCORE [4], QRISK [5, 6], and QRISK2 [7] are some well-known risk assessment studies that have offered highly useful recommendations for the prevention and treatment of cardiovascular diseases. CVD prediction models that target specific populations or integrate novel predictors have evolved in recent years to increase the models' predictive ability [8–10]. For example, Stuart et al. created a 10-year CVD risk prediction model for type 1 diabetic patients [9]. Emma et al. included the most common neuropsychiatric symptom of dementia, apathy, in a multivariate analysis for a CVD prediction model with people over 70 years old [10].

The pathogenesis of CVD is the result of a chronic vascular injury caused by a long-term interaction of multiple epigenetic factors of the body. Among these, blood pressure (BP) level is a major determinant of most CVD. Salt restriction is at the center of salt intervention studies aimed at preventing hypertension. However, there is no solid evidence that lowing sodium intake and increasing potassium intake can reduce the incidence of CVD [8–13]. In a prospective cohort examination of individuals from 17 countries, Martin O'Donnell et al. discovered that both greater and lower sodium consumption increased the risk of CVD [11]. On the other hand, Yuan et al. discovered that consuming more sodium and less potassium increased the risk of cardiovascular disease [12]. Our research examined whether a salt intervention (low-sodium salt or conventional salt) could be utilised as a predictor in a CV risk assessment model and whether low-sodium and high-potassium diets may affect the occurrence of CV events.

Several studies have demonstrated that genetic risk scores can improve the predictive value of conventional models, especially for people who are at moderate risk [13–16]. Genomic copy number variations (CNVs) are structural variations in DNA segments ranging in size from 1 kb to 3 Mb such as deletions, insertions, and duplications, and are a common kind of human genetic variation that affect the long stretches of DNA. CNVs affect a large number of chromosomes and have a high mutation frequency, resulting in large genetic variances in the population and the functioning of various characteristics. Researchers have discovered a link between lipoprotein(a) (LPA) CNVs and the risk of coronary heart disease (CHD) [17]. CNVs have also been demonstrated in other research to play a role in the aetiology of essential hypertension by influencing vascular resistance and energy metabolism, implying that CNVs may impact CVD development by affecting individual BP levels [18]. Previous studies have found that CNVs esv27061 and nsv483076 on chromosome 1 overlap with hypertension-related loci rs2932538 (Mov10 gene) and rs7129220 (ADM gene) respectively, suggesting that they may regulate BP levels by regulating smooth muscle contractility, vascular tone resistance, and water-sodium retention balance [18]. Therefore, CNVs esv27061 and nsv483076 were used to develop the model in this work.

In summary, this study looked at a group of people who had completed a three-year salt intervention and a ten-year follow-up in areas with a high incidence of hypertension. During the follow-up, researchers compared the differences in BP variation-related CNVs and other related characteristics between patients with and without CV events. Then, to offer a solid foundation for future customised interventions, a comprehensive analysis was done to analyse the effects of genetic and environmental factors on the long-term effects of CV events in the salt intervention cohort.

## Methods

### Study design and population

The previous study was conducted in two stages. In the first stage (intervention period, 2006–2009), salt intervention (low-sodium salt or conventional salt) was conducted by randomized controlled double-blind trial. Subjects who completed the first stage of intervention and had blood samples collected will enter into the second stage of the study. The second stage (follow-up period, 2009–2019) included a 10-year follow-up survey in which the subjects' outcome events were collected retrospectively. A detailed description of the two stages has been reported [19–21]. Basic information, medical history, physiology, blood biochemistry, and other CV risk variables were obtained via a questionnaire and physical examination in the prior study. In this investigation, the case group consisted of participants who had CV events in the previous study, whereas the control group consisted of people who did not have CV events. The nested case–control study approach was used to analyse the data. The CV outcome was defined as a composite of cardiovascular and cerebrovascular diseases, which were explored in detail below. Based on previous analysis, BP variation-related CNVs were discovered and used to screen out CV risk-associated CNVs loci. Then environmental and genetic information was then integrated to screen out the key evaluation indicators of CV events in the salt intervention population, and build a prediction model to assess the risk of CV events in this population.

Inclusion criteria for this study subjects: (1) completed the three-year intervention and had been followed up more than 6 times in the previous study; (2) could provide blood sample; (3) could sign the consent form. Exclusion criteria: (1) need to use potassium-sparing drugs (e.g., potassium-sparing diuretic) or potassium supplements; (2) had a history of obvious liver and kidney function damage; (3) withdrew or lost of follow-up in the previous study; (4) blood samples could not be used for BP variation-related CNVs detection. The study was authorised by the the First Affiliated Hospital of China Medical University's institutional review board. All surviving participants or relatives who provided information regarding deceased participants gave written informed consent.

### Outcome measurements

A combination of home follow-up, the death database, and the new rural cooperative medical care system (NCMS) was used to study the outcome measurements. The information on the commencement of CV events was gathered from surviving individuals by asking them directly, whereas the information from deceased subjects via asking their relatives. At the county level and above, the medical information had to be accompanied by a diagnostic certificate from the hospital, and the death from the household investigation had to be cross-checked against the death database. The composite events of cardiovascular and cerebrovascular diseases, encompassing disease onset and mortality, were designated as the outcome assessment of CV events. Each cardiovascular and cerebrovascular disease was assigned a code according to the International Classification of Disease, Tenth Revision (ICD-10) guideline, including heart diseases (Code: I05-I09, I11, I20-I27, I30-I52) and cerebrovascular diseases (Code: I60-I69).

### Risk factors

Potential risk factors included demographic factors, smoking, alcohol consumption, medical history, physiology, blood biochemistry, and others. Data collection methods included questionnaires, and physical and laboratory examinations. The questionnaire included age, sex, smoking (≥ 1 cigarette/d for ≥ 1 year), alcohol consumption (≥ 100 mL/d of Baijiu with > 50% alcohol content or ≥ 500 mL/d of beer for ≥ 1 year), history of hypertension (SBP ≥ 140 mm Hg and/or DBP ≥ 90 mm Hg, or taking medication of antihypertensive drugs or history of hypertension), history of CVD (whether had suffered from previous CVD), history of medication (whether using antihypertensive and other cardiovascular drugs), and salt intervention (low sodium salt or conventional salt). Physiological variables included height (cm), weight (kg), BP and heart rate, etc. The body mass index (BMI) was defined as the ratio of weight to height: BMI (kg/m^2^) = weight (kg)/height (m)^2^. Hospital staff collected and stored two 5 mL tubes of peripheral venous blood in a − 80 °C refrigerator. The blood samples were examined at the First Hospital of China Medical University Laboratory Center for serum triglyceride (TG), total cholesterol (TC), high-density lipoprotein (HDL-C), and low-density lipoprotein cholesterol (LDL-C). The remaining samples were used for BP variation-related CNVs testing.

According to published studies of Genome-Wide Association Study (GWAS) and CNVs analysis [18, 22], and searching the database for Genomic Variation (DGV, http://dgv.tcag.ca/dgv/app/home), [23] we selected the CNVs loci that may be related to the risk of human essential hypertension or CVD. The CNVs esv27061 and nsv483076 were the final loci identified in this investigation.

### Experimental materials and methods

Two selected CNVs loci were quantitatively detected by droplet digital PCR (ddPCR). Pre-experiments were performed with the OsciDrop® Flex digital PCR system and the template DNA to verify and optimize the suitability of the system and to determine the final concentration for testing.

1. Experimental materials

①template DNA; ②HS01327571 primer (Thermo Fisher); ③HS04399968 primer (Thermo Fisher); ④4403326 primer (Thermo Fisher); ⑤DNase&RNase free water (Beijing solarbio science & technology co., ltd.); ⑥DNA amplification system (Beijing Dawei biology technology co., ltd.); ⑦OsciDrop^®^ Flex digital PCR system.

(2) Experimental method

①Preparation of reaction solution following manufacturer protocol.

②After removing the bubbles from the solution, centrifuge it immediately.

③OsciDrop^®^ Flex digital PCR system was used to detect and analyze the results. Reaction temperature and time was set as follows: 95 ℃ 5 min; 95 ℃ 10 s; 59 ℃ 50 s, 45cycles; 25 ℃, ∞.

### Statistical analysis

The “MICE” package in R 4.1.3 was used to perform multivariate imputation of missing values [24]. Participants in both the case and control groups were statistically analysed. For each group, factors that may impact the risk of CV events were discussed independently. Continuous variables of normal distribution were described by the mean ± standard deviation ($\overline{X}$ ± S), and continuous variables that did not meet normal distribution were described by the median and interquartile range (M (IQR)). The statistical description of categorical variables was performed using percentages. Baseline characteristics between the case and control groups were compared by t-test for continuous variables of normal distribution, rank-sum test for continuous variables of non-normal distribution, and chi-square test for categorical variables. The Cox proportional hazard model was used for multivariate analysis, and collinearity diagnosis was performed to screen the independent risk factors affecting the outcome events. Set the terms for the interaction between the variables to be tested and time (time-dependent covariates). The violation was determined by the statistical significance of the Proportional Hazards Assumption (PH Assumption). The Akaike information criterion (AIC criteria) was used to calculate Cox regression for the highest amount of variation with the fewest possible independent variables. The “stepAIC” function of package “MASS” in R was used to screen variables, the direction of stepwise regression was “both”. The HR values and their 95% confidence intervals (95% CIs) were used to assess whether the indicators were associated with the outcome events as well as the strength and direction of the association.

Cox proportional hazard model was used to fit the survival data, and a prediction model including environmental and genetic data was constructed. The duration of survival was measured in months. The prediction model was evaluated from two aspects of discrimination and calibration. The Area Under Curve (AUC) was used to measure the discrimination of the prediction model. Considering the characteristics of survival data, a time-dependent ROC curve was used to assist in the diagnosis of discrimination. The receiver operating characteristic curve (ROC curve) was plotted with the true positive rate (sensitivity) as the ordinate and the false positive rate (1-specificity) as the abscissa. The cut-off value and its corresponding sensitivity and specificity were marked on the ROC curve, and the Youden's index (sensitivity + specificity−1) was calculated [25]. The AUC of the model above 0.7 was considered good discrimination. Brier score and calibration plot were used to measure the calibration of the prediction model. Brier score of the model between 0 and 0.25 was considered good calibration. The abscissa of the calibration plot was the predicted probability and the ordinate was the actual probability. Ideally, the calibration curve was a diagonal line (predicted probability equals actual probability). When the curve was below the diagonal line, the predicted probability was higher than the actual probability; conversely, it was less than the actual probability. The tenfold cross-validation method was used to internally validate the model. Interaction effects between independent variables were also considered. To explore whether the effect of BP on CV events was contingent on variations in CNVs, the interaction terms of two BP variation-related CNVs loci with baseline SBP and DBP were developed respectively.

Then we conducted sensitivity analysis to selection of alternative parameters. This prediction model was based on stepwise regression and AIC criteria, and was of professional significance. To quantify the improvement offered by CNV loci, we used net reclassification improvement (NRI) and integrated discrimination improvement (IDI) to examine the model performance. The risk stratification threshold of category-based NRI was 20% [26, 27]. If NRI > 0, the model performance has improved after adding CNV loci; if NRI < 0, the model performance has decreased; if NRI = 0, the model was considered not changed. IDI was judged in the same way as NRI.

The nomogram drawing with R was used to visualise the model. Using multivariate regression analysis, several predictors were combined, and calibrated line segments were drawn in a certain proportion on the same plane to indicate the link between variables in the prediction model. In the Cox model, each influencing factor was given a score depending on its contribution to the outcome variable. After then, the total score was obtained by adding all the scores together. Finally, the function conversion relationship between the overall score and the likelihood of the result event was utilised to calculate the predicted probability of each particular outcome event.

## Results

There were a total of 312 BP-related CNVs detected, including 8 in pre-experiments and 304 in the formal experiments. Among them, 287 cases of esv27061 were double copies and the remaining 25 cases (one copy or three copies) were copy number variation; 259 cases of nsv483076 were double copies and the remaining 53 cases (one copy or three copies) were copy number variation. In a prior study, 462 individuals were given the low-sodium salt or conventional salt intervention in 2006; 440 of them finished the intervention in 2009 and were followed for a decade. This study comprised 306 individuals who had completed the prior study and had blood samples taken, including 100 cases in the case group and 206 cases in the control group.

Table 1 shows the CNV frequency in the case and control groups. Table 2 (at the end of the document text file) shows the comparison of baseline characteristics between the case and control groups. There were statistically significant differences in age, smoking, history of hypertension, history of CVD, history of medication, baseline SBP, baseline DBP, TC, LDL-C, and CNVnsv483076 between the case group and the control group (*P* < 0.05).

**Table 1:** CNV frequency in the case and control groups

Number of copies	CNV^a^ esv27061			CNV^a^ nsv483076		
	1 copy (%)	2 copies (%)	3 copies (%)	1 copy (%)	2 copies (%)	3 copies (%)
Case group (%)	6 (6.0)	94 (94.0)	0	0	77 (77.0)	23 (23.0)
Control group (%)	15 (7.3)	187 (90.8)	4 (1.9%)	1 (0.5%)	179 (86.9)	26 (12.6)

^a^Copy number variation

**Table 2:** Baseline characteristics of the case and control groups

Characteristics	Case group	Control group	t/Z/χ^2^	*P*-value
N	100	206		
Age (years, M^a^ (IQR^b^))	55.00 (50.00–61.00)	44.00(35.75–53.25)	7.11	< 0.001
Sex			0.06	0.811
Male (n, %)	50 (50.00)	100 (48.54)		
Female (n, %)	50 (50.00)	106 (51.46)		
BMI^c^ [kg/m^2^, M^a^ (IQR^b^)]	27.42 (24.83–29.41)	26.56 (23.45–29.17)	1.82	0.069
Smoking (n, %)	51 (51.00%)	80 (38.83%)	4.07	0.044
Alcohol consumption (n, %)	39 (39.00%)	73 (35.44%)	0.37	0.544
History of hypertension (n, %)	94 (94.00%)	164 (79.61%)	10.54	0.001
History of CVD^d^ (n, %)	38 (38.00%)	21 (10.19%)	33.44	< 0.001
History of medication (n, %)	68 (68.00%)	75 (36.41%)	26.99	< 0.001
salt intervention (n, %)	49 (49.00%)	109 (52.91%)	0.41	0.521
Baseline SBP^e^ [mmHg, M^a^ (IQR^b^)]	164.25 (150.12–184.62)	148.00 (134.50–165.00)	5.03	< 0.001
Baseline DBP^f^ [mmHg, M^a^ (IQR^b^)]	95.50 (89.25–106.38)	90.50 (81.00–99.50)	4.08	< 0.001
Heart rate [M^a^ (IQR^b^)]	80.00 (71.25–89.00)	79.00 (72.00–87.25)	0.11	0.913
TG^g^ (mmol/L, M^a^ (IQR^b^))	1.44 (0.80–1.90)	1.16 (0.78–1.98)	1.11	0.265
TC^h^ [mmol/L, M^a^ (IQR^b^)]	5.52 (4.61–6.04)	5.00 (4.27–5.87)	2.52	0.012
HDL-C ^i^ [mmol/L, M^a^ (IQR^b^)]	1.55 (1.29–1.88)	1.62 (1.39–1.88)	0.52	0.601
LDL-C^j^ [mmol/L, $\overline{x}$ ± s]	2.67 ± 0.78	2.40 ± 0.74	2.95	0.003
CNV^k^ esv27061 (n, %)	6 (6.00%)	19 (9.22%)	0.93	0.334
CNV^k^ nsv483076 (n, %)	23 (23.00%)	27 (13.11%)	4.82	0.028

^a^Median

^b^Interquartile range

^c^Body mass index

^d^Cardiovascular diseases

^e^Systolic blood pressure

^f^Diastolic blood pressure

^g^Triglyceride

^h^Total cholesterol

^i^High-density lipoprotein cholesterol

^j^Low-density lipoprotein cholesterol

^k^Copy number variation

The variables included in the multivariable analyses were age (continuous variable), sex (man or women), BMI (continuous variable), smoking (yes or no), alcohol consumption (yes or no), history of hypertension (yes or no), history of CVD (yes or no), history of medication (yes or no), baseline SBP (continuous variable), baseline DBP (continuous variable), heart rate (continuous variable), salt intervention (low sodium salt or conventional salt), TG (continuous variable), TC (continuous variable), HDL-C (continuous variable), LDL-C (continuous variable), CNVs esv27061 and nsv483076 (yes or no). The results of multivariable analyses are shown in Additional file 1: Table S1. After the verification by PH Assumption and collinearity diagnosis, the variables were screened according to the AIC criterion, and the results of the multivariate Cox regression analysis are shown in Table 3. Aging, smoking, high LDL-C, high baseline SBP, CVD history, and CNV nsv483076 are all potential CV risk factors and included in the prediction model. Among them, aging, high LDL-C, CVD history, and CNV nsv483076 are all independent risk factors for future CV events (*P* < 0.05).

**Table 3:** The predictors included in the prediction model for CV events

	HR	95% CI		*P*-value
Age (years)	1.052	1.028	1.076	< 0.001
Smoking	1.443	0.962	2.166	0.076
LDL-C	1.372	1.031	1.824	0.030
SBP	1.008	0.999	1.017	0.078
History of CVD	2.021	1.306	3.127	0.002
CNV nsv483076	1.625	1.016	2.598	0.043

*LDL-C* low-density lipoprotein cholesterol, *SBP* systolic blood pressure, *CNV* copy number variation

The prediction model contained 6 potential major risk factors (age, smoking, LDL-C, baseline SBP, history of CVD and CNV nsv483076). The model formula was:

$$S({\text{t}}) =     {S_0}({\text{t}})\wedge\exp (0.050744983*{\text{age}} + 0.366860730*{\text{smoking}} + 0.315987955*{\text{LDL-C}} + 0.008091269*{\text{SBP}} + 0.703396717*{\text{CVD}} + 0.485283134*{\text{CNV\,nsv}}483076)$$

(age: continuous variable, years; smoking: categorical variable, 0 = no, 1 = yes; LDL-C: low-density lipoprotein cholesterol, continuous variable, mmol/L; SBP: baseline systolic blood pressure, continuous variable, mmHg; CVD: history of CVD, categorical variable, 0 = no, 1 = yes; CNV nsv483076: categorical variable, 0 = no, 1 = yes)

The predicted probability for the 13-year risk of CV events in this study was:

$$P\, = \,1 - 0.9975592\wedge\exp\left( {0.050744983*{\text{age}}\, + \,0.366860730*{\text{smoking}}\, + \,0.315987955*{\text{LDL - C}}\, + \,0.008091269*{\text{SBP}}\, + \,0.703396717*{\text{CVD}}\, + \,0.485283134*{\text{CNV\;nsv}}483076} \right)$$

**Fig. 1:**
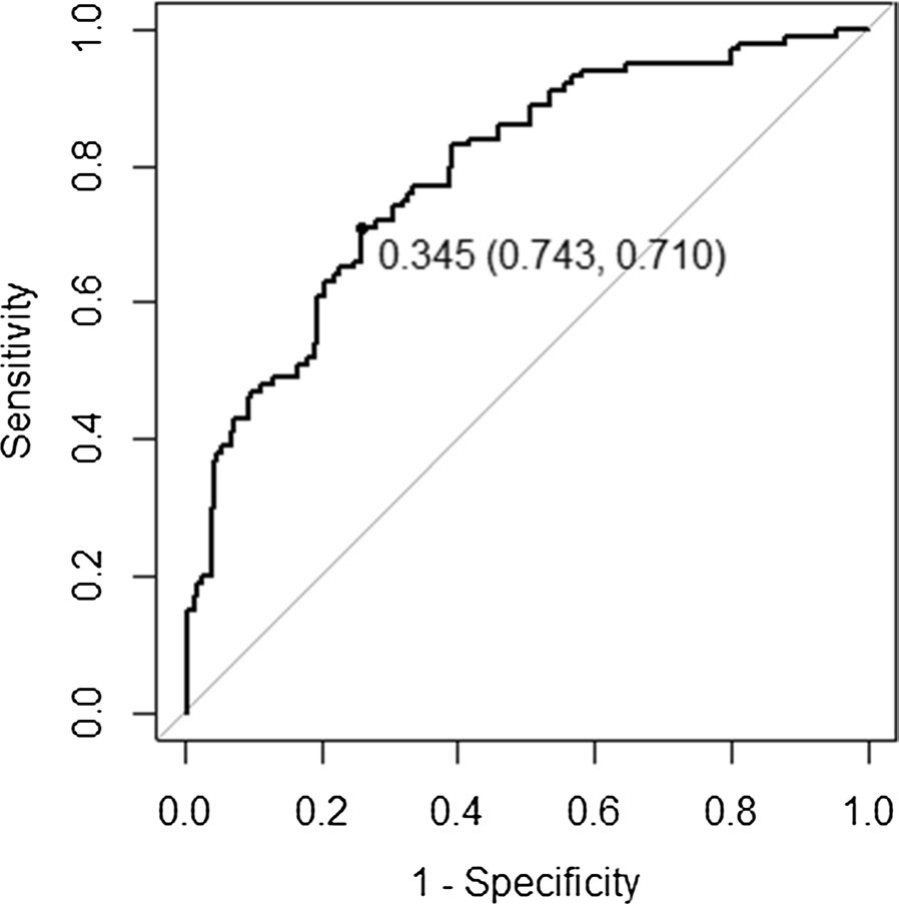
ROC curve for prediction model of CV events

**Fig. 2:**
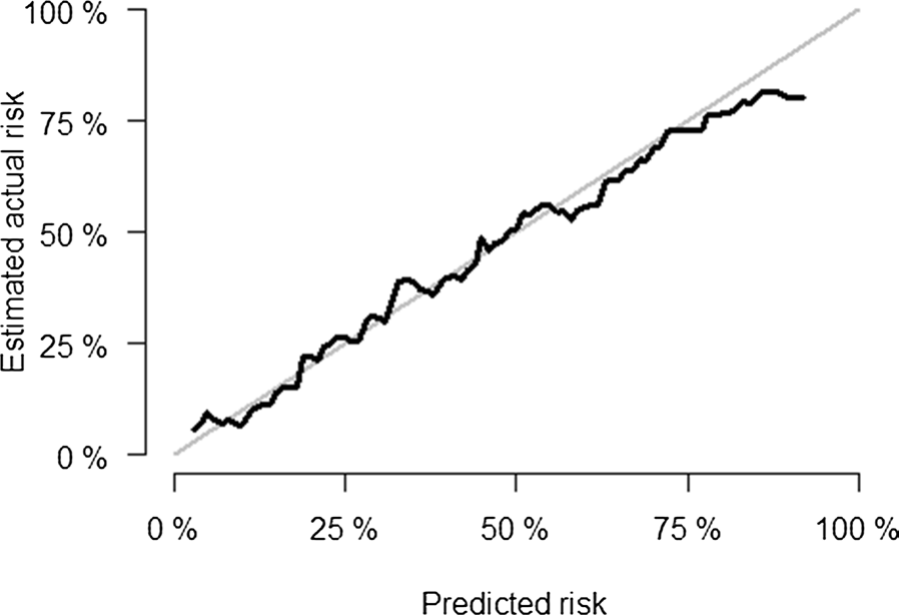
Calibration plot for prediction model of CV events

The risk stratification was conducted using a 20% cutoff point. If the expected probability for the 13-year risk of CV events was determined to be ≥ 20%, an individual was regarded to be at high risk, and if the predicted probability was calculated to be < 20%, they were thought to be at low risk. In this study, the AUC of the fitting model was0.788, and the time-dependent ROC curve was comparable, indicating acceptable model discrimination. The cut-off value of the ROC curve was 0.345, at this time, the sensitivity was 0.710, the specificity was 0.743, and the Youden’s index was 0.453. The Brier score of the model was 0.166, and the calibration plot showed that the projected probability was consistent with the actual probability, indicating acceptable model calibration. The ROC curve and calibration plot are shown in Figs. 1 and 2, respectively.

We also performed sensitivity analysis to quantify the improvement offered by CNV loci. And the following two models were established:

Model 1 contained variables: age, smoking, LDL-C, SBP, History of CVD;

Model 2 contained variables: age, smoking, LDL-C, SBP, History of CVD, CNV nsv483076;

Compared with model 1, the NRI of Model 2 was 6.4% (95% CI  − 2.7%, 11.8%; NRI + : 3.0%, NRI−: 3.4%), IDI was 0.9% (95% CI  − 0.8%, 3.8%). The results showed that the model performance was improved by adding CNV loci to the traditional model.

The model was internally validated using the tenfold cross-validation approach. In short, the dataset was randomly divided into 10 equal subsets with value allocation of 30, 31, 31, 31, 31, 31, 31, 31, 30, 30, and 30. One subset was used as the validation dataset while the other nine subsets were used as the training dataset. The AUC values for the 10 cross-validations were 0.6931217, 0.8750000, 0.7575758, 0.8229965, 0.7227273, 0.7013101, 0.7539301, 0.8666667, 0.8755981 and 0.8392857, with an average of 0.791. The Brier scores of 10 subsets were 0.1838332, 0.1209220, 0.1819830, 0.1506549, 0.2044108, 0.1787431, 0.1651426, 0.1795386, 0.1287678 and 0.1636890, with an average of 0.166. The model's internal validation was predicated on an average of ten validations, with an AUC of 0.791 and a Brier score of 0.166. The prediction model used in this investigation has high repeatability, according to the internal validation results. Table 4 shows the comprehensive findings of the tenfold cross-validation.

**Table 4:** The discrimination and calibration results of the tenfold cross-validation

validation dataset	AUC	Brier score
Fold-1	0.6931217	0.1838332
Fold-2	0.8750000	0.1209220
Fold-3	0.7575758	0.1819830
Fold-4	0.8229965	0.1506549
Fold-5	0.7227273	0.2044108
Fold-6	0.7013101	0.1787431
Fold-7	0.7539301	0.1651426
Fold-8	0.8666667	0.1795386
Fold-9	0.8755981	0.1287678
Fold-10	0.8392857	0.1636890
mean	0.791	0.166

**Fig. 3:**
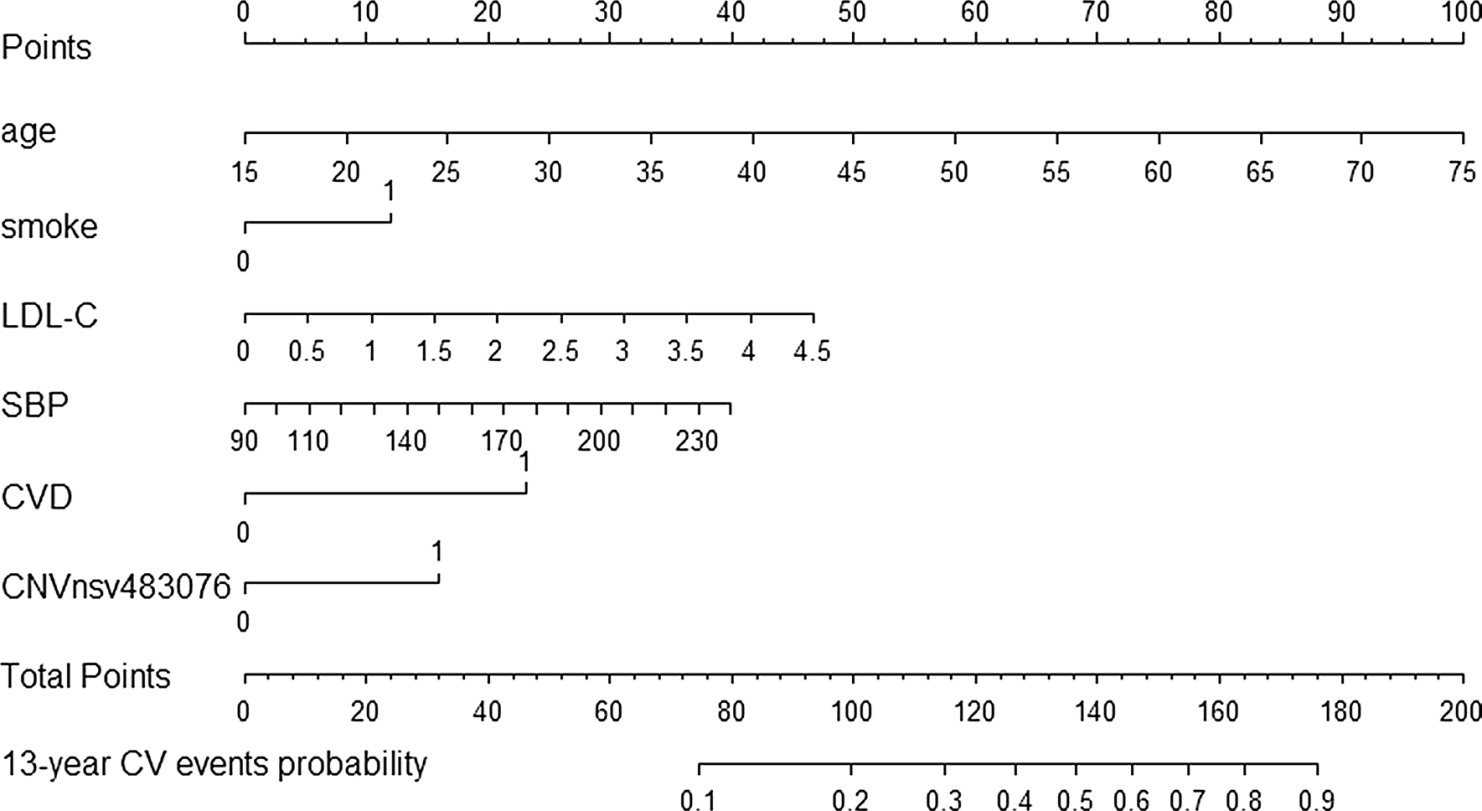
Nomogram of CV events prediction model. *LDL-C* low-density lipoprotein cholesterol, *SBP* systolic blood pressure, *CVD* cardiovascular diseases, *CNV* copy number variation, *CV events* cardiovascular events

As illustrated in Fig. 3, the prediction model was represented by a nomogram. The points, age (years), smoking, LDL-C (mmol/L), SBP (mmHg), CVD (whether had suffered from previous CVD), nsv483076, total points, and estimated probability for the 13-year risk of CV events were on the abscissa from top to bottom. An example was provided: a participant aged 45 years (score 50), smoking (score 12.5), LDL-C 2.7 mmol/L (score 27.5), SBP 165 mmHg (score 20), no history of CVD (score 0), CNV nsv483076 (score 16), the total score was 50 + 12.5 + 27.5 + 20 + 0 + 16 = 126, and the corresponding probability of CV events was 39%. Application of the CV events risk prediction formula:

$$\eqalign{
  P =  &  1 - 0.9975592\wedge\exp (0.050744983*45 + 0.366860730*1 \cr 
   &   + 0.315987955*2.7 + 0.008091269*165 + 0.703396717*0 \cr
   &  + 0.485283134*1) = 39.43\%  \cr} $$

The methodologies essentially agreed on the probability of CV occurrences, although the nomogram was simpler and more convenient than the prediction algorithm.

Finally, the model for screening variable included the interaction terms of two BP variation-related CNVs loci with baseline SBP and DBP, and nsv483076*SBP could be added into the model according to the AIC criterion. The model formula was list as follows:

$$\eqalign{
  S({\text{t}}) =  & {S_0}({\text{t}}) \wedge \exp (0.050684*{\text{age}} + 0.379350*{\text{smoke}} + 0.298358*{\text{LDL  -  C}} \cr 
  & + 0.011569*{\text{SBP}} + 0.683685*{\text{CVD}} + 3.493078*{\text{CNV}}{\mkern 1mu} {\text{nsv}}483076 \cr 
   &  - 0.018338*{\text{nsv}}483076*{\text{SBP}}) \cr} $$

This calculation yielded a negative regression coefficient, suggesting that the influence of baseline SBP on CV events may be greater when nsv483076 was normal double copies than when nsv483076 was copy number variation.

## Discussion

This study established a 13-year prediction model of the risk of CV events based on people in areas with a high prevalence of hypertension who had previously received a salt intervention. The prediction model included 6 risk factors: age, smoking, LDL-C, baseline SBP, CVD history, and CNV nsv483076. This work provided the risk prediction equation for a 13-year CV event, albeit the intricate regression equation was difficult to calculate in practice. As a result, we turned the equation into a visual graph to make the prediction model more understandable, simple, and practical. Furthermore, our modeling took into account the relationship between CNVs and baseline BP and found that the effect of baseline SBP on CV events may be larger when nsv483076 was normal double copies than when nsv483076 was copy number variation.

Our model contained six predictors, which is similar to many CVD prediction models reported in recent years [8, 10, 28, 29]. Johanna et al. performed a comprehensive assessment of CVD risk prediction models in the general population and discovered that most models included the same set of predictors, such as age, smoking, blood pressure, and blood cholesterol readings [30]. Our model includes age, smoking, LDL-C, and baseline SBP as predictors, which is similar to earlier CVD prediction models, implying that the model developed using these variables is reliable. We also stratified the anticipated probability risk of individuals. For those at high risk of CVD with a predicted probability value of ≥ 20%, recommended risk factor interventions such as smoking cessation, saturated fat reduction, and sodium reduction. Medication, such as anti-hypertensive medicines and lipid lowering medications, can be taken under the supervision of a clinician if necessary. The stratification of individual CV event risk is not absolute, and the thresholds for classifying high and low risk are not the same in different countries and regions. The risk stratification threshold is determined to match the actual onset of disease in a specific population and to realize the application value of the risk assessment model. In consideration of the high risk of CV events in people with a high prevalence of hypertension and the need to improve the sensitivity of disease screening, the cut-off value of 34.5% of the ROC curve was not selected as the threshold in our study. After selecting 20% as the threshold for risk stratification, the sensitivity reached 89%, which was 18% higher than the threshold of 34.5%. Of course, the risk values for the disease were continuously distributed in the population. We cannot simply assume that when an individual risk exceeds the threshold, some intervention should be initiated or clinical medication is required. Similarly, we cannot dismiss healthy lifestyle guidance as meaningless when the individual risk is below the threshold. We set a threshold of 20% for risk stratification according to the characteristic of specific population, in order to identify individuals at high risk of CV events as early as possible, improve their awareness, timely receive intervention or treatment, and make rational use of limited public health resources to obtain maximum benefits.

Genetic factors, as a lifelong risk factor, can help identify high-risk individuals early and lead to early lifestyle modifications, which are critical for the prevention and treatment of CVD. Using genome-wide polygenic scoring, researchers were able to identify 8% of the population as having a high risk of coronary artery disease (CAD), which was three times higher than the general population’s risk [16]. Single nucleotide polymorphisms (SNPs) have been introduced as predictors of genetic factors in CVD prediction models [31]. On the other hand, the total number of nucleotides covered by CNVs in the genome significantly outnumbers the total number of SNPs. Since their widespread distribution in the human genome was found, CNVs have been related to a number of diseases, including autoimmune diseases, infectious diseases, neuropsychiatric disorders, and CVD [17, 32–34]. Francine et al. evaluated all CNVs from a GWAS-based meta-analysis for genomic areas related to BP/hypertension to see if there were associated with essential hypertension or BP variance [22]. These CNVs were subsequently analysed using ddPCR, and the deletions esv2757747 on chromosome 1p13.2 and esv27061 on chromosome 1p13.2 were considerably higher in people with extreme hypertension, implying that CNVs were linked to essential hypertension in humans [18]. In this work, two CNVs loci were discovered and investigated using multivariate Cox regression analysis. CNV nsv483076 was found to independently increase the risk of CV events (P < 0.05) and was included in the final prediction model. Although we were unable to detect more CNVs loci due to sample size and financial constraints, our findings suggest that using genetic factors like CNVs as predictors allows the combined effects of non-genetic and genetic factors to be fully considered in modeling, resulting in improved model predictive performance.

Elevated BP is a risk factor for CVD, and high salt intake has been identified as one of the key dietary contributors in the development of essential hypertension. The study of the link between salt replacements and CVD has become a hot topic in recent years [21, 35, 36]. Neal et al. studied the effects of low sodium and high potassium salt substitutes on CV events in a 5-year cluster randomised trial in rural China [35]. In this trial, those who used salt replacements had a decreased risk of stroke and major CV events than those who used conventional salt. We discovered similar findings in a previous investigation [21]]. According to a 10-year follow-up study of patients who completed a three-year low-sodium or conventional salt intervention, low-sodium salt reduced the risk of CV death in adults with hypertension. However, the inclusion of salt intervention as a potential screening variable was not included in this study's prediction model. Due to the longer ten-year follow-up period compared to the three-year intervention period. Participants were unable to maintain their use of low-sodium salt after the intervention, limiting the advantage of reduced sodium intake.

The following are some of the advantages of this study: first, a novel predictor of BP variation-related CNVs was added to the old predictor, and a prediction model was created to elucidate the combined effect of genetic and environmental variables on the risk of CV events, improving the model's prediction performance. Second, regression equations were used to determine anticipated probability values for CV events risk, and probability thresholds for risk stratification were provided, which were more appropriate for practical applications and facilitated interventions in high-risk individuals. Third, the model was developed using a cohort of hypertension adults who were given a low-sodium or conventional salt intervention. The population's prediction performance was good, which was consistent with the recent trend of modeling for specific groups, and offered a foundation for future tailored low-sodium salt treatments. Fourth, the endpoint events were classified as a composite of cardiovascular and cerebrovascular disorders, which may have greater genetic heterogeneity than a single CVD, making it better suited to investigating changes in BP variation-related CNVs between patients. The study, however, had several flaws. First, due to a lack of external data, we were unable to externally validate the established model; nevertheless, we intend to gather adequate data for external validation in future research. Second, because long-term cohort studies were difficult to conduct, the sample size of our study was not sufficient to develop a sex-specific CV events risk prediction model. Third, the model was constructed for hypertensive people who were given low-sodium or conventional salt intervention. As a result, it cannot be applied to the broader public and cannot be universally applicable.

## Conclusions

After a three-year intervention with low-sodium salt or conventional salt and a 10-year follow-up, our CV events prediction model for patients living in high-hypertension areas functioned well. The model can be used to calculate the risk of CV events in specific populations by using environmental and genetic variables as predictors. It can help with fundamental CVD prevention and control, as well as act as the basis for personalized intervention strategies. We will require external samples in the future to further confirm our methodology and deliver risk assessments in specific demographics.

## Supplementary Information

**Additional file 1: Table S1.** The results of Cox multivariable analyses.

## Data Availability

The datasets generated and analysed during the current study are not publicly available due [reasons of sensitivity to human data] but are available from the corresponding author on reasonable request.

## Abbreviations

CVD: Cardiovascular disease
CV events: Cardiovascular events
AUC: Area under the curve
HDL-C: High-density lipoprotein cholesterol
LDL-C: Low-density lipoprotein cholesterol
SBP: Systolic blood pressure
DBP: Diastolic blood pressure
CNV: Genomic copy number variations
LPA: Lipoprotein(a)
NCMS: New rural cooperative medical care system
BMI: Body mass index
SNP: Single nucleotide polymorphisms
TC: Total cholesterol
TG: Triglyceride
GWAS: Genome-wide association study
DGV: Database for genomic variation
PCR: Polymerase chain reaction
PH Assumption: Proportional hazards assumption
AIC criteria: Akaike information criterion
CI: Confidence interval
ROC curve: Receiver operating characteristic curve
